# Reduction in Learning Rates Associated with Anterograde Interference Results from Interactions between Different Timescales in Motor Adaptation

**DOI:** 10.1371/journal.pcbi.1000893

**Published:** 2010-08-19

**Authors:** Gary C. Sing, Maurice A. Smith

**Affiliations:** 1 School of Engineering & Applied Sciences, Harvard University, Cambridge, Massachusetts, United States of America; 2 Center for Brain Science, School of Engineering & Applied Sciences, Harvard University, Cambridge, Massachusetts, United States of America; University College London, United Kingdom

## Abstract

Prior experiences can influence future actions. These experiences can not only drive adaptive changes in motor output, but they can also modulate the rate at which these adaptive changes occur. Here we studied anterograde interference in motor adaptation – the ability of a previously learned motor task (Task A) to reduce the rate of subsequently learning a different (and usually opposite) motor task (Task B). We examined the formation of the motor system's capacity for anterograde interference in the adaptive control of human reaching-arm movements by determining the amount of interference after varying durations of exposure to Task A (13, 41, 112, 230, and 369 trials). We found that the amount of anterograde interference observed in the learning of Task B increased with the duration of Task A. However, this increase did not continue indefinitely; instead, the interference reached asymptote after 15–40 trials of Task A. Interestingly, we found that a recently proposed multi-rate model of motor adaptation, composed of two distinct but interacting adaptive processes, predicts several key features of the interference patterns we observed. Specifically, this computational model (without any free parameters) predicts the initial growth and leveling off of anterograde interference that we describe, as well as the asymptotic amount of interference that we observe experimentally (R^2^ = 0.91). Understanding the mechanisms underlying anterograde interference in motor adaptation may enable the development of improved training and rehabilitation paradigms that mitigate unwanted interference.

## Author Summary

The act of learning one task can not only have direct effects on the performance of other tasks, but it can also affect the ability to learn other tasks. One example of the latter is the phenomenon of anterograde interference in motor adaptation, in which the learning of one adaptation can substantially reduce the rate at which the opposite adaptation can be learned. Here we show that the amount of anterograde interference depends systematically on the strength of a particular component of the initial adaptation rather than on the total amount of adaptation that is achieved. This component of the motor memory evolves more slowly than the overall learning and acts in combination with a quickly evolving component of memory to produce the observed improvement in task performance. We proceed to show that a simple computational model of the interactions between these adaptive processes predicts greater than 90% of the variance in the observed interference patterns, suggesting that this quantitative model may enable the development of improved training and rehabilitation paradigms that mitigate unwanted interference.

## Introduction

The history of prior action in the human motor system is known to influence not only future performance through memory, but also the capacity for future learning. Interference and savings are two oppositely-directed phenomena that produce this effect. Interference describes the ability of one task to impair the learning of another, while savings describes the ability of previous learning to enhance future learning. For example, previous work has shown that after initial learning and subsequent washout of a visuomotor rotation task, relearning is faster than the initial learning, even if the performance levels of the learner (i.e. the motor output) at the onset of learning and relearning are identical [1]–[2]. Similarly, in a saccadic gain adaptation task, after learning and subsequent opposite-learning such that the motor output returns to pre-learning levels, relearning is also observed to be consistently faster than initial learning [3].

Other studies have demonstrated that previous learning can hinder or interfere with future learning [4]–[10]. An experimental paradigm commonly used to study interference is the A_1_BA_2_ paradigm, where a subject is instructed to serially learn Task A, Task B, and then Task A again - often with various time delays inserted between tasks. In this paradigm, Task B is usually made to be the opposite of Task A (e.g. a clockwise vs. counterclockwise force-field or visuomotor rotation). Two types of interference can be studied with this paradigm – (1) retrograde interference: how Task B interferes with the memory of Task A_1_, and (2) anterograde interference: how the memory of Task A_1_ interferes with the subsequent learning of Task B (or how B interferes with A_2_). Note that both retrograde and anterograde interference can affect performance in Task A_2_.

Although anterograde interference can often have quite substantial effects [4]–[7], it has not received as much attention as retrograde interference in the motor adaptation literature. This is surprising because retrograde interference tends to have a relatively small (10–20%) effect on performance in the studies where it is reported [2],[11]–[13], whereas anterograde interference often has substantially larger effects [4]–[5]. In fact, several interference studies have been specifically designed to minimize the effects of anterograde interference because they recognized the potential it has for masking retrograde interference [2], [5]. Acquiring a better understanding of the mechanisms underlying anterograde interference is important not merely to provide greater insight into retrograde interference effects, but because the learning phenomenon is significant in and of itself as the primary cause of interference during motor adaptation.

Anterograde interference has been observed in force-field adaptation studies [4]–[5], [7] and visuomotor rotation studies [6], and has been shown to weaken as the time between tasks is increased [4]. A recently-proposed computational model for motor adaptation has suggested a possible mechanism for anterograde interference [14]. In this model, one internal adaptive process responds quickly to motor error, but rapidly forgets, while another adaptive process learns slowly from motor error, but has good retention. The contributions of these two processes are combined to generate the net motor output. In the transition from Task A to Task B, the “fast” process will quickly learn the new task, while the “slow” process will be reluctant to follow because of its good retention of the previous task. The multi-rate model predicts that the residual contribution of the slow process would hinder adaptation to Task B, resulting in anterograde interference. The model also predicts that as training in Task A is extended, the amount of interference will also increase, but then level off beyond 15–40 training trials in Task A. Here, using a simple AB paradigm to avoid retrograde interference effects, we examine for the first time how the duration of exposure to Task A influences the amount of anterograde interference observed in Task B in order to determine how the capacity for interference is built up with practice. We then use the predictions of the multi-rate model to determine whether anterograde interference stems from interactions between the different timescales of motor learning.

## Results

### Anterograde interference expressed as reduced force output

**Figure 1:**
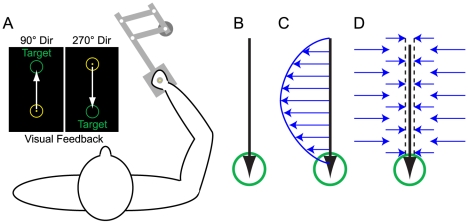
Illustration of experimental paradigm. *A*: Subjects grasped the handle of a robotic manipulandum while making 10 cm, point-to-point reaching motions in the 90° and 270° directions. Over the course of the experiment, subjects made three types of movements: (*B*) null field movements, where the robot motors were turned off, (*C*) force-field movements, where the robot applied a force pattern to the subjects' hands that was proportional to the reach velocity and perpendicular to the reach direction, and (*D*) error-clamp movements, where the robot acted like a spring/damper system in the direction perpendicular to movement(K = 6 kN/m, B = 250 Ns/m) such that 99% of lateral errors were clamped to 1.2 mm or less.

**Figure 2:**
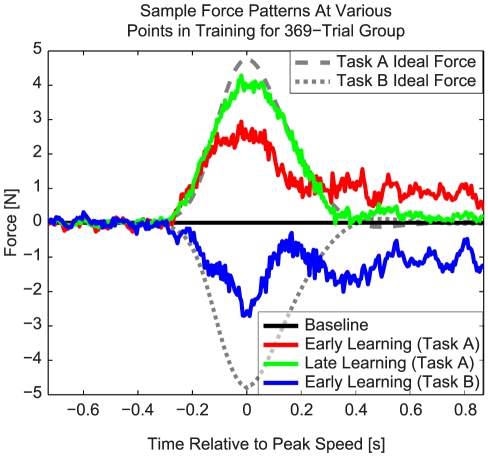
Force patterns at various points in training for the 369-trial group. The traces displayed here are the subject-averaged (n = 9 subjects) lateral force patterns produced during error-clamp trials at various points in training. All force patterns are baseline-subtracted. Gray dotted lines indicate ideal forces for Tasks A and B. Early learning force patterns (red) are taken from the first 25 trials of learning Task A. Late learning force patterns (green) are taken from the last 30% of trials while exposed to Task A (for the 369-Trial group, this corresponded to error-clamp trials interspersed throughout the last 95 force-field trials). Early opposite-learning force patterns (blue) are taken during exposure to Task B, and specifically are taken during the first 25 trials after subjects produced force patterns that most closely approximate baseline force production.

We studied how exposure to one motor adaptation task (Task A) influences the ability to learn a second task (Task B). It has previously been shown that prior exposure to Task A can induce anterograde interference in the learning of Task B when these tasks are opposite [4]–[7]. However, how the capacity for this interference builds up is unclear. Here we focused on how the duration of an initial motor adaptation to velocity-dependent dynamics (Task A) influences the amount of interference conferred onto subsequent adaptation to oppositely-directed velocity-dependent dynamics (Task B) during reaching arm movements (Figure 1A). We instructed different groups of subjects to learn clockwise [CW] (Figure 1C) or counter-clockwise [CCW] velocity-dependent force-fields for varying numbers of trials – either 13, 41, 112, 230, or 369 trials. After this initial exposure, subjects were switched to the opposite force-field (Task B) for about 115 trials (see Methods). Error-clamp trials were interspersed throughout the experiment (approximately 1 out of every 7 trials) to probe how the level of adaptation evolved during learning (Figure 1D; see Methods). Baseline-subtracted force patterns measured during these error-clamp trials at various points in training are displayed in Figure 2. Specifically, this figure shows the data averaged across subjects from the 369-trial group early and late in the training of Task A (early: red trace, average of first 25 trials; late: green trace, average of trials 259–369), and early in the training of Task B (blue trace, average of the first 25 trials after force output returned to baseline levels). Note that the force pattern produced during late learning of Task A closely matches both the magnitude and shape of the ideal force pattern, which would fully compensate the robot-imposed dynamics. The force pattern produced during early learning is, as might be expected, smaller in magnitude and less specific in shape. Early in training, the force pattern shows an appropriate transient component, but an inappropriate static component at the end of the movement. It has recently been shown that this static component arises because of a pervasive cross-adaptation between position-dependent and velocity-dependent dynamics [15]. Apropos to the current study, the force pattern produced early in Task B appears even smaller, suggesting the presence of anterograde interference from Task A onto Task B.

### Anterograde interference defined as a slower rate of learning

In this study, we define anterograde interference as the reduction in the learning rate for Task B due to previous learning of Task A. This definition is not entirely consistent with all previous work. Numerous studies have characterized anterograde interference by higher initial errors during the learning of Task B when compared to Task A [2], [6]–[7], [11]–[12], [16]–[18]. However, other work has defined anterograde interference in terms of slower learning of Task B instead [14], [19]. While these two definitions can sometimes be compatible, recent work has shown that this is not necessarily the case – higher initial errors (often associated with greater interference) can be coupled with faster learning rates, which indicates reduced interference [15]. Note that the sizes of initial errors have nothing directly to do with the ability to perform subsequent learning, as such. Initial errors in Task B (especially if these errors are in the feedforward component of motor performance) should instead reflect the continuity of performance from the end of Task A, in particular when Task B immediately follows Task A [4]–[7]. When a time delay is inserted between these two tasks [4], initial errors for Task B reflect the retention of Task A. Thus, interference defined this way may say more about performance levels achieved in Task A than the extent to which Task A interferes with the ability to learn Task B.

**Figure 3:**
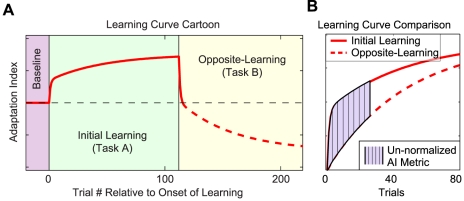
Schematic of anterograde interference metric. *A*: A cartoon example of a learning curve in an AB learning paradigm, where Task A (green background) is learned prior to Task B (yellow background). The dashed line represents the portion of the learning curve during Task B after the baseline performance level is achieved. Note that in this cartoon example, opposite-learning proceeds more slowly than the initial learning because of the previous exposure to Task A. We define this slowing as anterograde interference. *B*: To quantify the extent to which anterograde interference slows adaptation to Task B, we compute the percent reduction between the initial learning curve for Task A (solid red line) and the rectified (flipped) opposite-learning curve for Task B starting from when the adaptation achieves baseline levels (dashed red line) over the first 25 trials of these learning curves. This analysis specifically evaluates the reduction in learning rate rather than higher initial errors, because in the comparison both learning curves start from the same overall performance level.

**Figure 4:**
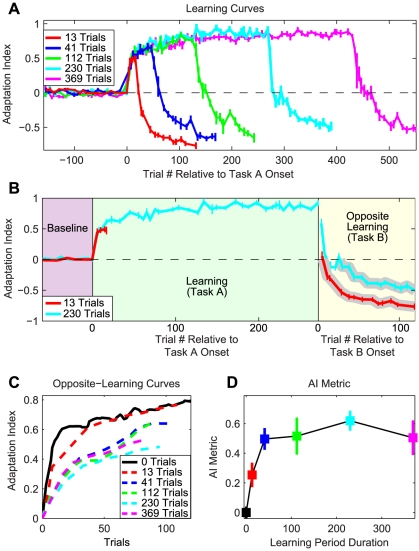
Experimental data from anterograde interference learning paradigm. *A*: All subjects performed 160 null baseline trials, and then learned Task A (either a CW or CCW velocity-dependent force-field) for varying durations, followed by Task B (a CCW or CW velocity-dependent force-field, respectively). Learning curves are averaged across subjects. *B*: Learning curves for the 13-trial (red curve) and 230-trial groups (cyan curve) aligned to task onset. The portions of the opposite-learning curves displayed in C and analyzed in D are represented by the gray-highlighted regions. *C*: Opposite-learning curves proceeding from the zero-crossing point for all groups, smoothed with a 3-pt moving average. See FIG. S8A for the raw opposite-learning curves. *D*: The anterograde interference metric, calculated as shown in FIG. 3B, increases as the duration of the initial learning period (Task A) increases; however, after 15–40 learning trials, interference asymptotes. In all panels, errorbars represent SEM.

In order to dissociate the interference conferred from Task A onto Task B from the performance level achieved in Task A, we focus on the learning rate observed in Task B once baseline performance has been achieved. Specifically, we compare the opposite-learning curve for Task B (i.e. the rectified response to Task B starting from when the net adaptation crosses zero; Figure 3B, dashed red line) to the initial learning of Task A (Figure 3B, solid red line), and use the percent reduction in the Task B learning curve as a metric of interference (see Methods and Figure 3). If the opposite-learning curves are aligned at the zero-crossing, as illustrated in Figure 4C, then the initial learning and opposite-learning curves will start from the same performance level (i.e. zero learning), and the Task B learning rate can then be directly compared to the Task A learning rate. If the learning curves are compared from task onset rather than zero-crossing, the patterns of performance are similar, regardless of whether anterograde interference occurs (see Text S1, Figure S5 and Figure S6). Note that this comparison between initial and subsequent learning curves proceeding from the same performance level is analogous to the comparison between initial learning and relearning rates in the analysis of savings. In the analysis of data from savings experiments, if the unlearning is not complete, initial performance during the second learning period reflects retention of the first adaptation, which is difficult to disambiguate from faster relearning [1], [3], [19].

### Learning curves for Task A-Task B paradigm

We quantified adaptation levels by regressing the actual force patterns like those displayed in Figure 2 onto the ideal force patterns for each task (see Methods) [20]. We refer to the slope of this regression as the adaptation index for a particular trial. Perfect compensation for the force-field would yield an adaptation index of 1, while no learning would yield an index of 0. Group-averaged learning curves based on these adaptation indices are shown in Figure 4A (see Text S1 for an analysis of the R^2^'s for these regressions (Figure S1), as well as for alternative methods for estimating the regression slopes (Figure S2 and Figure S3)). Adaptation can also be assessed by quantifying the amount of force associated with learning-related changes used to counteract the force-field. Since the lateral force required to oppose the force-field is greatest at the peak speed point, which is near the middle of the movement, we used the average mid-movement force as a secondary measure of the progression of adaptation. Here we define mid-movement force as the average force produced during a 250ms window centered at the movement's peak speed. These data are displayed in Figure S4A. We found that both the regression coefficients and mid-movement force metrics revealed learning curves which were essentially identical in shape to one another. In agreement with previous studies [5], [12], [14]–[15], [20]–[25], we found that the adaptation to the initial velocity-dependent force-field (Task A) is at first rapid, and then more gradual. However, upon exposure to Task B, we consistently observed alterations in learning curves that indicated the presence of anterograde interference: after the initial unlearning of Task A brings the learning curves back to zero (the baseline adaptation level), the opposite-learning (learning of Task B) appears to proceed more slowly than the initial learning. Figure 4B shows a more direct comparison of Task A and Task B learning curves for the 13-trial and 230-trial groups. The portions of the opposite-learning curves proceeding from the zero-crossings are highlighted with a gray background. Note that this portion of the opposite-learning curve is slower for the 230-trial group than the 13-trial group, consistent with the presence of increased anterograde interference.

### Relationship between interference and duration of Task A

Analysis of the opposite-learning curves displayed in Figure 4C clearly illustrates the presence of anterograde interference. All of the opposite-learning curves are slower than the initial learning curve, illustrated as the nominal 0-trial group. The opposite-learning curves based on the mid-movement force data also show this effect (Figure S4B). We created a best overall estimate of the initial learning curve by aggregating the data from the initial learning curves from all five groups. The learning curves presented in Figure 4C are smoothed with a three-point moving average (for non-smoothed versions of these curves based on regression coefficients, see Figure S8). We defined a metric for the amount of anterograde interference caused by initial adaptation to Task A by computing the percent reduction in the opposite-learning curves (with respect to initial learning) over the first 25 trials (Figure 3B).

We found that the duration of exposure to Task A had a significant effect on the amount of interference (one-way ANOVA, F(5,76) = 14.87, p = 2.7×10^−10^), indicating that as exposure to Task A is increased from 0 trials, the amount of interference significantly increases. All of the groups experienced significant interference when compared to the aggregated initial-learning curve (Figure 4B; one-tailed, unpaired student t-tests, p-values between 2.8×10^−9^ and 1.4×10^−3^). Direct comparison of each group's initial-learning and opposite-learning curves reveals that this significant interference is present for all groups, and not just in the comparison with the aggregated initial-learning curve (i.e. opposite-learning curves are significantly slower than the initial-learning curves within each group; one-sample, one-sided student t-tests, p-values between 2.8×10^−6^ and 0.02; because the 13-trial group did not complete 25 trials in Task A, we compared initial and opposite-learning over the first 13 trials in that case). However, we found no significant differences between the interference metrics observed for the 41-trial, 112-trial, 230-trial, and 369-trial groups (one-way ANOVA, F(3,32) = 0.38, p = 0.77), but *did* find a difference when we included the 13-trial group (one-way ANOVA, F(4,45) = 2.71, p = 0.042), indicating that the increase in interference levels off after 15–40 trials (Figure 4D) at a value of about 0.53. Interference metrics calculated using the mid-movement force data follow the same pattern as those calculated using the regression coefficients (Figure S4C).

### The pattern of anterograde interference is explained by a multi-rate learning model

**Figure 5:**
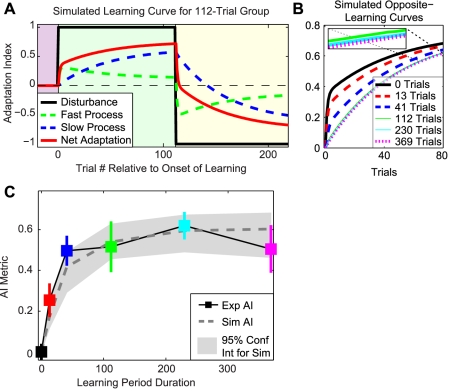
Response of multi-rate model to anterograde interference learning paradigm. *A*: In response to Task A (disturbance = +1), the fast process quickly learns, leading to rapid initial net adaptation. As the error in the system decreases, the fast process begins to forget more than it gains, leading to the non-monotonic shape of the fast process over the course of Task A. At the same time, the slow process gradually increases in strength and eventually becomes the main contributor to the net adaptation. This leads to the more gradual rise in adaptation level observed later in Task A. At the transition to Task B (disturbance = −1), the fast process quickly responds to the sudden change in error by diving below zero. However, the slow process is initially biased against learning Task B. The combination of this initial bias and the perseveration of this process results in slower net adaptation to Task B - i.e. anterograde interference. *B*: Comparison of the predicted, rectified opposite-learning curves for the different groups aligned at the zero-crossing point, consistent with the data analysis shown in Figure 4. Note that the amount of anterograde interference increases substantially over the first 41 trials, but that the opposite-learning curves for the 112-trial, 230-trial, and 369-trial groups are very similar to each other - the inset shows a zoomed-in version of these curves. *C*: Comparison of experimentally-observed and model-predicted levels of anterograde interference for all groups. Note that the experimental data displayed here is identical to that shown in Figure 4D. The parameters used to generate the model predictions for anterograde interference (dark gray dashed line) were not found by fitting the parameters to the current data set - instead they were taken from a previous study [14]. The 95% confidence intervals (light gray shaded region) for the simulation predictions were determined from 1000 different fits to bootstrapped versions of this previous data set (see Methods). Errorbars represent SEM.

What can explain the observation that increasing exposure to Task A leads to greater interference when adapting to Task B, but that this increase in interference then eventually asymptotes? One possibility is that this pattern of interference results from interactions between different components of the adaptive process. A recent study has suggested that a simple two-process, multi-rate learning model might explain several key features of motor adaptation as a consequence of predictable interactions between these two processes [14]. This learning model is composed of a “fast process,” which learns very quickly but forgets quickly, and a “slow process,” which learns slowly but has good retention. The contributions of these two processes are combined to generate the net motor output. The learning curves predicted by this model for the AB adaptation paradigm studied in the current work are displayed in Figure 5A. Note that none of the parameter values we used for this model (see Methods) were fit to the current data set; rather, all parameter values were taken from a data set in a previous study (which looked at spontaneous recovery rather than anterograde interference) [14]. Ideal performance for Task A is represented as an adaptation index of +1, while Task B is represented as −1.

Initially, the overall learning (red curve) is rapid because the fast process (green curve) quickly responds to the motor error. However, this rapid learning results in a quick decrease in the amount of error driving the learning. As a result, the amount of learning decreases and the fast process begins to forget more than it learns, leading to a decline in its level beginning around 10–20 trials after the onset of learning. In parallel, the slow process (blue curve) gradually increases in level, and eventually becomes the main contributor to overall learning around 25 trials after exposure to Task A begins. When Task B is presented, the fast process quickly responds because of the increase in error magnitude. However, the slow process follows much more gradually. The external state (overall learning) returns to baseline levels (an adaptation index of zero) when the fast and slow processes are equal in magnitude but opposite in sign. Note that at this point, although the external state is at baseline levels, the internal states do not match their baseline levels. The residual positive bias of the slow process (see blue curve in Figure 5A at task transition) acts to retard the opposite-learning of Task B (ideal performance = −1). The longer that Task A is learned, the greater the level the slow process achieves, leading to greater anterograde interference, as illustrated in Figure 5B. However, note that if Task A is learned for longer than is required to achieve asymptotic adaptation in the slow process, increasing exposure to Task A should not lead to a corresponding increase in interference.

We simulated the multi-rate model's response to an AB learning paradigm for Task A durations of 13, 41, 112, 230, and 369 trials and a Task B duration of 115 trials (i.e. the task durations used in the experiment). By comparing the predicted opposite-learning curves for these different groups (Figure 5B), it becomes apparent that increasing the duration of Task A exposure leads to slower opposite-learning curves – all of the opposite-learning curves are slower than the 0-trial group, which is identical to the initial-learning curve for Task A. However, the predicted opposite-learning curves for the 41-trial, 112-trial, 230-trial, and 369-trial groups are extremely similar, resulting from similar levels of the slow process during Task A between trials 41 to 369. When we quantify the amount of interference predicted for each group (Figure 5C, gray dotted line), we found a close match to the experimental data (Figure 5C, colored squares). Note that this match is not the result of model fitting because the parameters of the multi-rate model used to generate these predictions were taken from previous work in which anterograde interference did not occur [14].

The degree to which a model accounts for data is often characterized by a correlation coefficient or, equivalently, the corresponding R^2^ value derived from a two degrees-of-freedom (DOF) linear regression (slope and offset) of the relationship between the model output and the data. This regression yields an R^2^ value of 0.93 (regression slope = 0.89, offset = 0.05). However, the idea of an offset parameter implies that anterograde interference will exist even if Task A is not trained. As this is an unreasonable implication, we could restrict the linear regression to just one DOF (the slope). In so doing, we find that the multi-rate model is able to characterize the measured pattern of interference with an R^2^ value of 0.91 (regression slope = 0.992). Note, however, that the multi-rate model should not merely predict the shape of the interference pattern, but the actual levels of interference. Thus, when we abandon the regression altogether and directly compare the model predictions and experimental data, we find that our ability to explain the data remains essentially the same, with an R^2^ value of 0.91. This suggests that anterograde interference results from interactions between the different timescales of motor learning.

### Final learning level hypothesis cannot explain anterograde interference

Although the data presented so far appear to be consistent with the predictions of the multi-rate learning model, they are also consistent with the idea that the level of motor output at the end of Task A is what actually dictates the amount of anterograde interference. For example, in Figure 4B, the final level of motor output for the 230-trial group is higher than that for the 13-trial group (one-tailed unpaired student t-test, p<3.2×10^−6^): note that adaptation coefficients of 0.86±0.03 and 0.47±0.05 observed in these two groups correspond to lateral force production levels of 3.8±0.1N and 2.2±0.3N (mean±SEM), respectively (see Figures 4A, 4B, and Figure S4A; we operationally define final learning as the last 30% of Task A exposure, see Methods). The 1.6N increase in motor output displayed by the 230-trial group might explain why this group experiences more interference than the 13-trial group. To evaluate this hypothesis, we instructed an additional group of subjects to learn a 50% reduced Task A (i.e. the force-field strength was halved to 7.5 Ns/m) for 230 trials, followed by a switch to a full-strength Task B. Given that perfect performance during this reduced Task A would correspond to an adaptation index of 0.5 and a mid-movement force level of less than 2.5N (see Methods), and that subjects achieve about 85% of perfect learning (Figure 4A), corresponding to an adaptation index of 0.43 based on the full-strength force-field, the final learning level would be expected to be less than the final adaptation level of the 13-trial group (0.47±0.05, mean±SEM).

**Figure 6:**
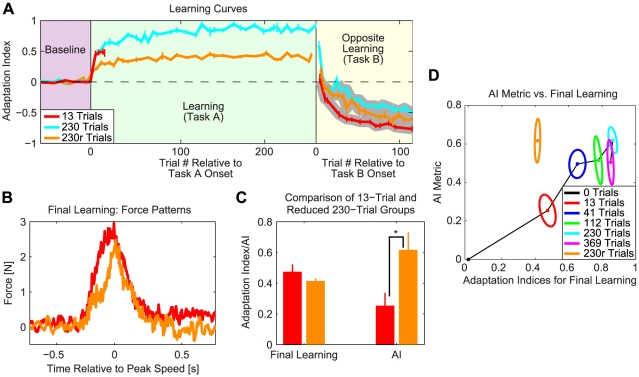
Evaluation of the final learning level hypothesis. *A*: Learning curves for the 13-trial (red curve), 230-trial (cyan curve), and reduced 230-trial (orange curve) groups. The reduced 230-trial group were exposed to a half-strength (B = 7.5 Ns/m) Task A, and then a full-strength (B = −15 Ns/m) Task B. *B*: Subject-averaged force patterns during final learning (last 30% of Task A trials) for the 13-trial (red) and reduced 230-trial (orange) groups. *C*: The reduced 230-trial group displays significantly higher levels of anterograde interference than the 13-trial group (*p<0.03), despite nominally lower levels of final learning. This finding is inconsistent with the hypothesis that the amount of interference depends on the level of final learning. *D*: Relationship between final learning and anterograde interference for all groups. Errorbars and confidence ellipses represent SEM in panels A, C, and D.

The learning curves for the full 13-trial, full 230-trial, and reduced 230-trial groups are shown in Figure 6A. As expected from the experimental design, the final learning level for the reduced 230-trial group (0.41±0.02, mean±SEM) is nominally less than the final learning level of the 13-trial group (Figure 6C; two-tailed unpaired student t-test, p = 0.18). Correspondingly, when comparing the mid-movement force levels, we see that the reduced 230-trial group produces significantly less force than the 13-trial group (1.5±0.1 N vs. 2.2±0.3 N, respectively; p<0.04, two-sided unpaired student t-test; Figure S4D). The lateral force patterns observed at the end of Task A for these two groups reflect this difference (Figure 6B). Therefore, if the final learning level hypothesis were true, it would predict that this reduced 230-trial group would experience *less* interference than the 13-trial group, corresponding to *faster* opposite-learning. However, as shown in Figure 6A, the shaded portion of the opposite-learning curve for the reduced 230-trial group is *slower* than its 13-trial group counterpart. Accordingly, the reduced 230-trial group experiences significantly *more* interference than the full 13-trial group (Figure 6C: p<0.007, one-tailed unpaired student t-test on regression data; Figure S4D: p<0.007, one-tailed unpaired student t-test on mid-movement force data) despite smaller learned changes in motor output at the end of Task A (p<0.04, as mentioned above). This finding is not consistent with the final learning level hypothesis. Figure 6D, which displays the interference metric plotted against the final learning for all of the groups, highlights this inconsistency. Therefore, while the amount of anterograde interference displayed by the full strength force-field groups could be interpreted as being dependent on final learning levels because they all lie along the same line, the reduced 230-trial group cannot because it is separated from this line. This is not to say, however, that the final learning level is completely independent of the amount of AI expressed. According to the multi-rate model, both Task A duration and Task A strength influence the level of the slow process, which according to the multi-rate model is the ultimate determinant of AI (see Figure S11).

## Discussion

We examined how the motor system's capacity for anterograde interference builds up with practice by studying how the amount of interference from Task A onto Task B varied with the amount of training on Task A. We found a gradual build up in the amount of interference that reached asymptote after about 15–40 trials. Interestingly, we found that the amount of anterograde interference is not directly related to the performance level achieved in Task A (see Figure 6). Specifically, we found that the reduced 230-trial group expressed significantly more interference than the 13-trial group, even though this reduced group displayed lower levels of final learning. However, we found that the relationship between the amounts of interference we observed and the duration of the Task A training period is accurately predicted by a model of the interactions between two adaptive processes with different learning rates, as is the asymptotic level of anterograde interference. This suggests that anterograde interference results from interactions between the different timescales of motor learning. This model had been previously applied to explain experimental data for the shapes of initial learning curves [14], [20], [25], adaptation deficits in patients with cerebellar damage [26], different patterns of memory consolidation [24], patterns of spontaneous recovery [14], [25], and levels of 24-hour retention in motor adaptation [20]. Here we used parameters that were taken from previous work in order to avoid the issues associated with curve-fitting so that this model's predictions were not constrained by the current data set in any way. We found that this model accurately predicted both the shape (R^2^ = 0.93) and the actual levels (R^2^ = 0.91) of interference observed in the relationship between the amount of training in Task A and the level of interference onto Task B.

### Alternative explanations for anterograde interference

We showed that the observed pattern of anterograde interference cannot be explained by a model relating anterograde interference to the level of motor output at the end of Task A (see Figure 6). However, anterograde interference has also been proposed to arise from a delay in switching between different internal models [4], [12]. This explanation could be interpreted in two different (and opposite) ways. On one hand, a delay in switching could be a qualitative description of the residual positive bias of the slow learning process once it slowly begins to adapt to Task B. In this case, our findings would not only support this delayed switching explanation, but provide a quantitative mechanism for it. On the other hand, if we interpreted the delayed switching explanation as meaning a delay in switching between *any* internal models, then such a mechanism would appear to be at odds with the rapid improvement in performance that can occur under conditions that produce savings. For example, a saccadic gain experiment using an A_1_BA_2_ learning paradigm found *faster* relearning of Task A during the second exposure, even though this second instance of Task A immediately followed exposure to Task B [3], and thus would require a switch between different internal models. If delays did exist when switching between different internal models, then one would expect *slower* relearning of Task A. While this second interpretation cannot account for the previous work on savings in the A_1_BA_2_ paradigm, our multi-rate model can [14]. Furthermore, it can explain the measured pattern of anterograde interference reported in the current work, rapid downscaling and unlearning of a learned motor task [27], 24-hr retention of a motor task [20], the shapes of initial learning curves, adaptation deficits in patients with cerebellar damage [26], patterns of memory consolidation [24], and spontaneous recovery in force-field [14] and saccadic gain adaptation [25]. See Text S1 and Figure S9 and Figure S10 for a discussion of other possible alternative explanations for anterograde interference.

### Saturation of learning

Interestingly, the amount of anterograde interference we observed in the reduced-230 group (0.62±0.11, mean±SEM) was significantly higher than what was predicted by the multi-rate model (0.33, p = 0.03, one-sample, two-sided student t-test; Figure S11). Perhaps this discrepancy can be explained by the observation that the motor system may process larger errors differently from smaller errors [28]. For example, in a force-field adaptation task, retention is better when the force-field is gradually introduced (i.e. small errors) than if it is abruptly introduced with larger errors [29]. This finding suggests that the level of the slow process is elevated when adapting to small errors, yielding better retention (which is equivalent to reduced decay) because the slow process would decay more slowly than the fast process. In keeping with this idea, it would be likely that adaptation to the reduced-strength Task A is composed of a greater-than-expected contribution from the slow process because of the smaller errors during training, thus leading to greater-than-expected interference.

How might this be achieved mechanistically? Greater-than-expected levels of the slow process when adapting to smaller errors could be achieved if learning in the two internal processes (i.e. 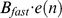, 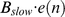) saturates as errors increase in size, and if the slow process saturates *earlier* than the fast process. Stated in another way, elevated levels of the slow process would manifest if the ratio 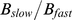
were higher for smaller errors in the linear region than for large errors in the saturated region. This occurs only if 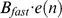 is still rising as error increases when 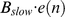 has already saturated, resulting in an increased gap between the learning rates in favor of the fast process.

This is in contrast to our current model, which assumes a fully linear relationship between learning and error (i.e. 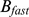 and 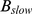 are constant over the space of all possible errors). However, it is important to note that no biological system remains linear over all space, and evidence suggests that, in fact, motor adaptation saturates as errors get larger. For example, when subjects are exposed to increasingly strong force pulses during reaching arm movements, single-trial adaptation levels saturate even though the kinematic errors induced by these transient force perturbations steadily increase [30]. This indicates that as errors increase beyond a certain point, single-trial learning levels saturate, and learning rate decreases. Similarly, when subjects are exposed to visual feedback shifts during arm reaches, small errors induce essentially linear adaptation, but larger errors lead to saturation of single-trial learning, or even a decrease in learning for extremely large errors [31]. An even more striking example of the nonlinearity of learning with respect to error can be found in saccadic gain adaptation [32]. When monkeys are exposed to small visual errors while making eye saccades, adaptation is linear. Over an intermediate range of errors, adaptation saturates, as previously discussed. However, when errors are increased past this intermediate range, motor output actually falls back to near baseline levels, indicating that the decrease in learning rate associated with these very large errors is more than enough to counteract the benefit of a larger learning signal. While saturation clearly occurs in the learning process as errors get larger, further work is required to confirm whether the slow process does indeed saturate earlier than the fast process. If this occurs, it may simultaneously explain why gradual adaptation to a force-field with small errors leads to better retention than abrupt adaptation with large errors [29], and why we see greater-than-expected interference in our reduced-strength 230-trial group.

### The multi-rate model is sufficient to explain anterograde interference, but not savings

A recent visuomotor rotation study showed that savings occurs in an A_1_-washout-A_2_ relearning paradigm, although the amount of savings observed is less than in an A_1_BA_2_ relearning paradigm [1]. In this paper, the authors use a superposition argument to show that a linear multi-rate model cannot yield savings in the A_1_-washout-A_2_ paradigm, suggesting that the savings observed in the A_1_BA_2_ paradigm cannot be fully explained by interactions between internal adaptive processes. They suggest that some (nonlinear) memory of Task A or a meta-learning process may also contribute to savings. In contrast, in the current work, we show that interactions between adaptive processes appear to fully account for the observed pattern of anterograde interference (R^2^ = 0.91). The multi-rate model predicts a near-asymptotic interference level of 0.54 for the four longest duration full-strength groups on average, which closely matched the average interference level we observed for these groups (0.53).

### Neural correlates of adaptive processes

It has been shown that patients with cerebellar deficits are significantly impaired in their ability to learn new motor skills [26],[33]–[35]. Since a reduction in adaptation is evident after just a few trials, these findings suggest that the cerebellum might be a neural substrate for the fast process. However, even if the fast process were more affected than the slow, the dramatic reductions in motor adaptation observed in these studies suggest that both processes are likely to be affected by cerebellar damage [26], [35].

Recent work has shown that while application of transcranial magnetic stimulation (TMS) to the posterior parietal cortex (PPC) does not impair the rapid initial learning of a viscous force-field, it does eliminate the gradual increase in learning that the multi-rate model attributes to the slow process [23], suggesting that the slow process might depend on the PPC. Findings from several other studies indicate that primary motor cortex may also serve as a neural substrate for the slow process. These studies have shown that stimulation of primary motor cortex may cause a partial reduction in the retention factor of the slow process. For example, in a visuomotor rotation task, when TMS is applied to primary motor cortex immediately after movement offset, adaptation to the rotation is unaffected, but this adaptation washes out more rapidly when the visuomotor rotation is withdrawn [36]. Interestingly, the more rapid washout only emerges after the third or fourth trial, suggesting that the retention of the slow, but not the fast, process might be preferentially impaired by this TMS. Another study found that 24-hour retention of adaptation to a viscous curl force-field is reduced by about 15% if a 15-minute block of repetitive (1Hz) TMS is applied to primary motor cortex prior to the onset of training [37]. Since 24-hour retention is specifically determined by the level of the slow (and not fast) process at the end of training [20], this finding also suggests that stimulation of primary motor cortex can result in a partial reduction of the retention factor of the slow process.

Consistent with these results, neurophysiologic data recorded from primary motor cortex during force-field adaptation reveal the existence of “memory cells” that retain adaptive shifts in preferred direction, even after the behavioral signs of adaptation have been washed out [38]. The activity of these memory cells is consistent with what would be expected from the output of the slow process, which is responsible for anterograde interference. This interpretation should be taken with some degree of caution, however, because reanalysis of the same data suggested that the tuning curves of neurons in primary motor cortex may drift spontaneously [39].

### The ABA paradigm and retrograde interference

In an A_1_A_2_ learning paradigm, a reduced initial error and a faster learning rate can be observed in adaptation to Task A_2_ compared to A_1_, even when prolonged time periods (such as a day or week) separate A_2_ from A_1_ [2], [5], [11], [20]. When a second task (Task B, which is often taken to be the opposite of Task A) is inserted between A_1_ and A_2_ in the A_1_BA_2_ paradigm, improvement on Task A_2_ can be reduced. This reduction has been attributed to the ability of Task B to erase, in whole or in part, the memory of A_1_ [2], . This effect is known as retrograde interference because Task B interferes with a previously-stored memory. Complete retrograde interference from Task B onto the retention of Task A_1_ has been reported when only 5 minutes separate the two tasks [2], [5], [11]–[12], [22], [40]–[42]. However, if 4 to 24 hrs separate A_1_ and B, then retrograde interference can be reduced, reflecting the consolidation of the initially fragile memory of Task A_1_ into a more stable form [2], [11]–[12].

Intriguingly, one recent study found complete interference of Task B onto A_2_ for both 5 min and 24 hr intervals between A_1_ and B in visuomotor rotation and force-field adaptation paradigms [5], in contrast with the finding that a 24 hr interval after Task A_1_ is sufficient for either partial or full consolidation [2], [12]. A series of studies have attempted to reconcile the differences between these findings by suggesting that the inclusion of “catch trials” (occasional movements during which the learned environment was unexpectedly removed) [13], or washout trials before Task A_2_ (null-field trials to wash out contributions from anterograde interference which could mask retrograde interference effects in Task A_2_) [2] are necessary for consolidation to be observed. However, even these proposals do not provide a fully harmonious explanation for all of the available data: consolidation has been observed even when catch trials are not included [2], and two experimental conditions with the null-field movements to washout anterograde interference failed to show evidence for consolidation, even with a 24hr interval between Tasks A_1_ and B [5].

Although the weight of the evidence suggests that consolidation can occur during motor adaptation, the somewhat fragmented nature of these results indicates that the mechanisms governing this resistance to retrograde interference are not yet fully understood. This may be substantially due to the fact that retrograde interference has a relatively small (10–20%) effect on performance in all of these studies [11]–[13], making resistance to retrograde interference somewhat challenging to study. In contrast, the effects of anterograde interference can be substantially larger [4]–[5]. In the current study, we found that anterograde interference reached levels of 50–60%, suggesting that anterograde interference can play a substantially greater role in modulating the rate of motor learning than retrograde interference.

It is interesting to note that when washout trials are not included before Task A_2_ to prevent anterograde interference, performance on Task A_2_ has been reported to be similar to naïve performance on Task A_1_ in studies of visuomotor rotation [2], [6]. It has been suggested that this occurs because anterograde interference from B onto A_2_ effectively cancels the performance improvement conferred by the memory of A_1_ [6]. An alternative hypothesis is that Task B interferes with the ability to retrieve the memory of A_1_ [2], [19]. This idea is consistent with the observation that the performance on Task A_2_ and A_1_ are similar, even when one week separates B and A_2_ – a time period long enough for aftereffects of B to have minimal influence on performance of Task A_2_ [2], [5], and consistent with a mechanism for interference with retrieval posited for declarative memories [43]–[44] . This mechanism can be viewed as a type of hybrid between anterograde and retrograde interference because it describes a forward (anterograde) effect of Task B, but the effect impairs retrieval of the memory for the previously-learned (retrograde) Task A_1_. Note that our multi-rate model would not, by itself, be able to explain such a mechanism.

### Multiple timescales

Intriguingly, as exposure to Task B progresses, we find that the opposite-learning curves for the different groups (Figure 4C) do not converge to the degree predicted by our two-rate model (Figure 5B). This discrepancy could potentially be explained by the existence of slower learning processes with even more protracted timescales than the “slow” learning process in the model we applied. The residual contributions from these slower processes could hold back adaptation to Task B for even longer, leading to even slower convergence. While it is likely that more than two timescales are present in motor adaptation [45], it is remarkable that interactions between just two adaptive processes are able to predict the shapes of initial learning curves, anterograde interference, savings, rapid downscaling and unlearning of a learned motor task, 24-hr retention of a motor task, and spontaneous recovery of learning [3], [14], [20], [25], [27].

## Methods

### Participants and ethics statement

Fifty-eight healthy individuals (34 women, median age: 24 yrs old, age range: 18–64, 52 right-handed) participated in this study. Each of the subjects had no prior knowledge of the experiment's purpose and provided informed consent. All experiment protocols were approved by the Harvard University Committee on the Use of Human Subjects in Research.

### Task

Participants were given a dynamic force-field adaptation task to learn [46]. They were asked to sit in front of a vertically-mounted computer screen while grasping the handle of a two-joint robot arm manipulandum (Interactive Motion Technologies, Inc.) that allowed motion in the xy-plane (Figure 1A). The xy-position of the handle was indicated by the xz-position of a cursor (3 mm in diameter) on the computer screen. Subjects were instructed to make 500 ms, 10 cm reaching arm movements in the y-direction (in the midline, toward or away from the chest) from one circular target (10 mm in diameter) to another in as straight a line as possible. Although subjects made movements in both the 90° and 270° directions, only movements in the 270° direction were analyzed; all 90° movements were “error-clamped” by using the robot arm as a virtual spring (6 kN/m) and damper (250 Ns/m) [14]–[15], [20]–[21], [47] such that the maximum lateral deviation from a straight line connecting the start and end targets during the longitudinal reach motion was 1.2 mm (Figure 1D).

Initially, subjects were asked to make 160 reaching movements in the 270° direction during a baseline training period. Approximately 90% of these baseline trials were made while the manipulandum's motors were turned off (Figure 1B). The other 10% of trials were error-clamp trials, during which lateral errors were restricted to no more than 1.2 mm. With maximal lateral kinematic errors about 1% of the reach length of 10 cm, online kinematic error feedback contributions to motor output are mostly eliminated, such that the measured force production is composed of predominantly feedforward contributions [14]–[15], [20]–[21], [24], [29], [48]. Following this baseline period, subjects were then exposed to a velocity-dependent force-field environment (Task A) for a variable number of trials in the 270° direction (13-trial group: 14 subjects; 41-trial group: 9 subjects; 112-trial group: 9 subjects; 230-trial group: 9 subjects; 369-trial group: 9 subjects). In this viscous force-field, the manipulandum imposed forces onto the hand that were perpendicular to the reach direction and proportional to the reach velocity (Figure 1C):
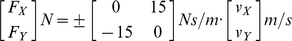
(1)In order to move in a perfectly straight line while being perturbed by the force-field, subjects would need to produce a compensatory force pattern that exactly counteracts the robot-produced force. Half of each experiment group experienced a clockwise force-field during Task A. The other half experienced an equal magnitude counter-clockwise force-field. Following the completion of Task A, subjects were then exposed to the opposite force-field (i.e. if Task A was a clockwise force-field, Task B was a counter-clockwise force-field) for about 115 trials (116, 114, 113, 112, 120 trials for the 13-trial, 41-trial, 112-trial, 230-trial, and 369-trial groups, respectively). Interspersed throughout Tasks A and B were occasional error-clamp trials (approximately 1 out of every 7 trials) in order to assess the learning curve associated with learned feedforward force output produced by subjects.

By measuring lateral forces during error-clamp trials during this force-field adaptation task, we can assess how well the magnitude and shape of subjects' force outputs compare to the ideal velocity-dependent force pattern, which is the opposite of the robot-produced force (Figure 1C). We regress the subject-produced force pattern onto the ideal force pattern in order to quantify the learning – an absence of any learning would yield a regression coefficient (or adaptation index) of 0, while perfect learning would yield an adaptation index of 1. Note that an index of 1 does not necessarily mean that subjects produced perfect compensatory forces. The actual force pattern can be decomposed into a component that is aligned with the ideal force pattern, and a component that is orthogonal to it. The regression coefficient indicates the size of the aligned component and is independent of the orthogonal component. If the regression coefficient is 1, this indicates that the magnitude of the aligned component is exactly the ideal force profile, regardless of the size of the orthogonal component. We use these adaptation indices to generate the learning curves displayed in Figures 4, 6, and Figure S8. These adaptation indices are then averaged across subjects to obtain group-averaged data. Note that at the onset of Task B, the ideal force pattern becomes opposite of that required in Task A, whereas the force patterns being produced are still appropriate for the Task A ideal force. Therefore, at the transition from Task A to Task B, the regression coefficients jump from one value to the negative of that value (e.g. for the 369-trial group, the coefficients jump from the adaptation level at the end of Task A (0.85) to −0.85, Figure 4A). To maintain continuity in the adaptation curves plotted in Figures 4A, 4B, and 6A, we multiply the regression coefficients calculated during Task B by −1, which is equivalent to maintaining a consistent ideal force pattern throughout the duration of the plot. Therefore, the negative values observed in these adaptation curves late in exposure to Task B reflect the fact that subjects are producing motor output that is nearly equal to the ideal output for Task B, and nearly opposite the ideal output for Task A. Also note that the “rectified” opposite-learning curves shown in Figure 4C actually represent the “original” regression coefficients, equivalent to multiplying by −1 *twice* (once in the manipulation just described, and once in the rectification).

It is possible that the two-rate behavior we observe in the current paper is a result of averaging together two sub-populations of subjects with different single-rate behaviors. However, we observed interference even when comparing individual initial and opposite-learning curves to each other (i.e. opposite-learning curves are significantly slower than the initial-learning curves within each group; one-sample, one-sided student t-tests, p-values between 2.8×10^−6^ and 0.02; because the 13-trial group did not complete 25 trials in Task A, we compared initial and opposite-learning over the first 13 trials in that case).

Note that the subject-produced force patterns we present are baseline-subtracted, where the baseline is the average force pattern measured during the last 5 error-clamp trials before the onset of Task A. Subjects who first learned a CCW force-field and then a CW force-field had their force patterns multiplied by negative one so that their data could be aligned with the subjects who first learned the CW and then CCW force-field. All force and velocity profiles used in the analysis were 2.25 seconds long and centered at the peak longitudinal velocity point (where longitudinal velocity refers to the component of the velocity vector in the target direction). We also used a mid-movement force metric defined as the average force produced during a 250ms time window centered at the peak velocity point. The data for this force metric can be found in Figure S4. Note that the force levels associated with this metric would be expected to be somewhat smaller on average than the force levels observed right at the peak velocity point.

An additional group (8 subjects) learned the same Tasks A and B as the 230-trial group, except that the force-field in Task A was halved in magnitude:
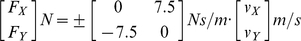
(2)See the Results section for the reason why this additional group was studied. The adaptation indices for this reduced-strength group were calculated by comparing the subject-produced force patterns to the full-strength ideal patterns for Tasks A and B in order to allow for comparison with the full-strength groups.

### Two-process, multi-rate learning model

We recently proposed a two-process, multi-rate learning model that provides a potential explanation for anterograde interference, along with several other motor learning phenomena [14]. The model states that a force disturbance of the motor system introduces a motor error that drives the evolution of two constituent learning processes that have different rates of learning and retention. This motor error is the difference between the overall motor output (i.e. the combined contributions of the two processes) and the desired output necessary to compensate for the force disturbance.
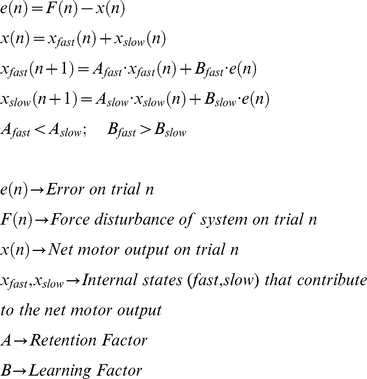



One of these processes, 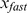, learns quickly from error, but rapidly forgets the previous learning. The other process, 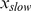, learns slowly from the error, but retains what it previously learned very well. This occurs because, 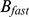 is greater than 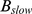, and 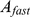 is less than 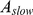, leading to multiple timescales in the learning process. The values for these parameters, 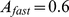, 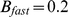, 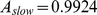, and 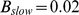, were taken from a previous study [14] in which anterograde interference did not occur rather than being fit to the current data set. See Text S1 for model parameters fit to the current data set.

### Anterograde interference metric

We calculate the percent reduction in the opposite-learning curves for Task B with respect to the initial-learning curve for Task A in order to quantify the level of anterograde interference. Specifically, we use the portions of the opposite-learning curves beginning from the zero-crossing point (i.e. when the performance level has returned to baseline levels) in this analysis and rectify them such that comparisons with the initial learning curve can be made directly. This percent reduction, or anterograde interference metric, is measured over the first 25 trials because the difference between the curves is greatest early on (Figure 3). A metric value of 0 corresponds to no interference, and a metric value of 1 corresponds to a complete lack of opposite learning, or 100% interference.
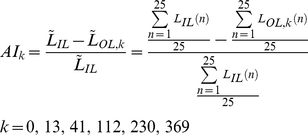
(3)

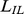
 represents the average initial learning between trials 1 and 25, and 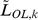 represents the average opposite learning within the same boundaries, and *k* indicates the subject group. Note that we interpolate between trials 1 and 25 for the initial-learning and opposite-learning curves to find the average learning. In addition, note that because this average learning is proportional to the area under the curves over that same trial span, a normalized AI metric based on average learning (Equation 3) is identical to a normalized AI metric based on area under the curve which is illustrated in Figure 3B. 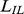 was found by combining the initial-learning curves for all subjects exposed to a full-strength force-field in Task A. If after task transition the learning curve crossed zero, went back above zero, and then crossed zero again (as in the 230-trial and 369-trial groups), we used the last zero-crossing as the beginning of the opposite-learning curve. See Text S1 and Figure S7 for a discussion of our rationale for choosing to use this particular AI metric, as opposed to the time constant of the opposite-learning curves.

To compare the experimentally-obtained interference metrics with simulation predictions, we found 1000 different sets of model parameters by bootstrapping previously-obtained data [14] and calculating the associated interference metrics predicted by the multi-rate model. We then found the 95% confidence interval for the simulation predictions by selecting the metrics representing the 2.5% and 97.5% percentiles as the interval boundaries.

### Data analysis

We use one-tailed, paired t-tests to compare the initial-learning and opposite-learning between groups, using the average interpolated learning between trials 1–25, with the exception of the 13-trial group. Because the initial-learning period for this group only spans 16 trials (13 force-field trials, 3 error-clamp trials), we compare the learning between trials 1–16.

The Average Final Learning metric (Figure 6) is obtained by averaging together the force patterns measured during the error-clamp trials in the last 30% of trials during initial learning, corresponding to trials 9–13, 28–41, 78–112, 161–230, and 259–369 for the 13-trial, 41-trial, 112-trial, 230-trial, and 369-trial groups, respectively.

## Supporting Information

Figure S1 R^2^ values associated with regressions.*A*: Learning curves of regression coefficients. These curves are averaged across subjects. *B*: R^2^ values associated with the regression coefficients in panel A. These curves are also averaged across subjects. In both panels, errorbars represent SEM.
(0.45 MB EPS)

Figure S2 Different methods for estimating regression slope. Displayed are learning curves for the 369-trial group using different methods of regression. These regressions are: (1) the standard *y*-onto-*x* regression which we use in the main text (blue curve), (2) the reciprocal of the slope found by the *x*-onto-*y* regression (red curve), (3) the Deming regression (black), and (4) a *y*-onto-*x* regression, where *y* is the first principal component of the actual force pattern, and *x* is the first principal component of the ideal force pattern (green). Note that the plot window limits are restricted because the *x*-onto-*y* regression leads to occasional instabilities associated with dividing by the near-zero values of force present in the baseline and early training data.
(0.29 MB EPS)

Figure S3 Different methods for estimating regression slope for averaged force patterns. The solid-line curves displayed here are the slopes calculated from regressions of force patterns averaged across subjects for individual trials, whereas the dashed blue line is calculated from regressions of force patterns on individual trials for individual subjects, and then averaged across subjects. These curves are for the 369-trial group using different methods of regression. These regressions are: (1) the standard *y*-onto-*x* regression which we use in the main text (blue curves), (2) the reciprocal of the slope found by the *x*-onto-*y* regression (red curve), (3) the Deming regression (black), and (4) a *y*-onto-*x* regression, where *y* is the first principal component of the actual force pattern, and *x* is the first principal component of the ideal force pattern (green). Note that the plot window limits are restricted in order to facilitate distinguishing between the different traces.
(0.30 MB EPS)

Figure S4 Mid-movement force duplicates regression analysis. *A*: Instead of using regression coefficients as adaptation indices for our learning tasks, we used the mid-movement force as a proxy for learning. We define mid-movement force as the average force produced during a 250ms window centered on the peak speed point of a movement (i.e. the average force produced from −125ms to +125ms; see FIG. 2 for examples of these force patterns). Learning curves here are averaged across subjects. *B*: Opposite-learning curves proceeding from the zero-crossing point for all full-strength groups, smoothed with a 3-pt moving average. *C*: Comparison of experimentally-observed and model-predicted levels of anterograde interference for all full-strength groups. The anterograde interference metrics are calculated using either the regression coefficients (solid squares, black line) or mid-movement force (empty squares, maroon dotted line, shifted 10 trials to the right to facilitate viewing). Both patterns are very similar to each other. Note that the data corresponding to “Exp AI (reg)” is identical to that shown in Figures 4D and 5C. The parameters used to generate the model predictions for anterograde interference (dark gray dashed line) were *not* found by fitting the parameters to the current data set - instead they were taken from a previous study (Smith et al. 2006). The 95% confidence intervals (light gray shaded region) for the simulation predictions were determined from 1000 different fits to bootstrapped versions of this previous data set (see Methods). *D*: The reduced 230-trial group displays significantly higher levels of anterograde interference than the 13-trial group (p<0.02, one-tailed unpaired student T-test), despite significantly lower levels of final force production (p<0.02, one-tailed unpaired student T-test). This finding is inconsistent with the hypothesis that the amount of interference depends on the level of final learning or force production. Errorbars in all panels represent SEM.
(0.55 MB EPS)

Figure S5 A single-process model does not predict interference. A single-process learning model with learning coefficient B = 0.03 and retention coefficient A = 0.9923 does not predict any anterograde interference. *A*: Red trace is learning during Task A and the portion of Task B training *prior* to the zero-crossing. The black dashed trace is the opposite-learning following the zero-crossing point. *B*: Red trace is initial learning of Task A. Black dashed trace is the rectified opposite-learning curve starting from the zero-crossing point.
(0.32 MB EPS)

Figure S6 Comparison of AI metrics starting from Task B onset and zero-crossing point. *A*: The raw adaptation level after 50 trials of exposure to Task B. Colored squares are data, dotted line is the single-process prediction, dashed line is the two-process prediction, and the gray shaded region is the 95% confidence interval for the two-process prediction. *B*: The change in adaptation between the beginning of Task B and after 50 trials of exposure to Task B. Gray lines and regions are the same as in panel A. *C*: The average-learning based interference metric used in the main text.
(0.33 MB EPS)

Figure S7 Time constant as an interference metric. *A*: Time constants of the opposite-learning curves for each group, averaged across subjects. Linear *y*-scale. *B*: Time constants of the opposite-learning curves for each group. Log *y*-scale. *C*: AI metric used in main text (FIG. 4D, 5C). *D*: Coefficient of variations for the time constant metric and the average-learning metric. Dotted gray line is the average coefficient of variation for the time constants, and the dashed gray line is the average coefficient of variation for the average-learning metric. All error bars are SEM.
(0.36 MB EPS)

Figure S8 Possible choices for zero-crossing point. *A*: If, after task transition, the net learning curve went below zero, rose above it, and went below zero again, we chose to use the last zero-crossing point as the beginning of the opposite-learning curves. Only the 230-trial and 369-trial groups were affected by this choice. The simulated pattern of anterograde interference explained the experimental pattern of anterograde interference well (R^2^ = 0.91). *B*: Using the average of the first and last zero-crossings (when the learning curve is positive before the zero-crossing and negative afterwards) as the zero-crossing also yields a close correspondence between the experimental and simulated patterns of anterograde interference (R^2^ = 0.86).
(0.38 MB EPS)

Figure S9 Memory trace framework simulation. *A*: Simulation of the memory trace framework learning an AB learning paradigm. Solid red curve is from Task A onset to the zero-crossing point after Task B onset. The dashed red curve is the opposite-learning curve starting from the zero-crossing point. *B*: Comparison of the initial-learning curve (solid red curve) to the rectified opposite-learning curve (dashed red curve).
(0.32 MB EPS)

Figure S10 Exponential vs. power law decay. Decay as a function of trial number. The red curve is the decay predicted by the two-exponential, multi-rate model, while the blue curve is the power law decay predicted by the memory trace framework model.
(0.28 MB EPS)

Figure S11 Effect of Task A duration and the magnitude of Task A perturbation on AI metric. Magnitude of Task A perturbation is given in terms of percentage of full-strength perturbation of 100%, or a force-field strength of 15 N-s/m. Color mapping indicates level of AI metric, and is proportional to *z*-level value.
(0.51 MB EPS)

Text S1 Supporting information. Contains further discussion about R^2^ values of the regressions for adaptation indices, alternative methods for estimating regression slope, mid-movement force as an adaptation index, alternative anterograde interference metrics, possible choices for the zero-crossing point, alternative explanations for anterograde interference, the effect of Task A duration and magnitude of Task A perturbation on the AI metric, and the multi-rate model parameters fit to the current data set.
(0.18 MB PDF)
