# Supplementary material for: Reduction in Learning Rates Associated with Anterograde Interference Results from Interactions between Different Timescales in Motor Adaptation
Source: PLoS Comput Biol. 2010 Aug 19;6(8):e1000893. doi: 10.1371/journal.pcbi.1000893 (PMC2924244; doi:10.1371/journal.pcbi.1000893)
Supplement: Text S1 — Supporting information. Contains further discussion about R2 values of the regressions for adaptation indices, alternative methods for estimating regression slope, mid-movement force as an adaptation index, alternative anterograde interference metrics, possible choices for the zero-crossing point, alternative explanations for anterograde interference, the effect of Task A duration and magnitude of Task A perturbation on the AI metric, and the multi-rate model parameters fit to the current data set. (0.18 MB PDF) [file pcbi.1000893.s012.pdf]

<u>TEXT S1</u>

R^2 values of the regressions for adaptation indices

The R^2 values associated with the regressions used to calculate the adaptation indices are displayed in Figure S1B. Note that the actual force patterns being regressed can be decomposed into a component that is aligned with the ideal force pattern, and a component that is orthogonal to it. The regression coefficient indicates the size of the aligned component and is independent of the orthogonal component (Figure 4A and S1A). In contrast, the R^2 value depends on both the aligned and orthogonal components. Specifically, the R^2 value is the ratio of the variance explained by the aligned component compared to the total variance (total variance = aligned variance + orthogonal variance). Note that when the adaptation indices approach zero (Figure S1A), the R^2's also approach zero because the aligned components are getting smaller while the orthogonal components are not, and *not* because the signals inherently become noisier. After the zero-crossing points, the adaptation indices increase in magnitude, and this increase in signal is reflected in the "rebound" in the R^2 coefficients.

Alternative methods for estimating regression slope

In our regression analysis, we regress the actual force pattern (*y*) onto the ideal force pattern (*x*). Since both of these time-series are noisy, regressing *x* onto *y* will in general yield a different result than regressing *y* onto *x* because regressions are biased by noise in *x* (Berkson 1950). This bias can be addressed with a *Deming regression analysis*, which provides an unbiased estimate of the slope by minimizing both *x*-errors and *y*-errors simultaneously, but in a weighted fashion (Cornbleet and Gochman 1979, Linnet 1998, Wakkers et al. 1975). Practically speaking, the unbiased Deming slope lies in between the biased slopes calculated by the two standard regressions (i.e. *y* onto *x*, and the reciprocal of the slope found by regressing *x* onto *y*), and the weighting between these two slopes is effectively determined by the ratio between noise in the two signals. For example, if *y* is much noisier than *x*, the Deming slope will be much closer to the *y*-onto-*x* slope, which is less biased because of the smaller amount of noise in *x*. The practical problem with performing the Deming correction is that noise levels in *x* and *y* can be difficult to estimate accurately, which is why in some situations it may be preferable to use a standard *y*-onto-*x* or *x*-onto-*y* regression. However, we attempt to estimate levels below.

The blue trace shown in Figure S2 is the learning curve calculated from the standard *y*-onto-*x* regression (i.e. actual force onto ideal force). The reciprocal of the other standard regression (*x*-onto-*y*) is shown as the red curve. Note that we are restricting our plot window limits because the *x*-onto-*y* regression leads to occasional instabilities associated with dividing by the near-zero values of force present in the baseline and early training data. In order to perform the Deming regression, we need to estimate the ratio of noise in the two signals. Within a given trial, noise can come from two different sources: (1) measurement noise and (2) intrinsic motor variability. We estimated the variances as:

Noise Source	Measurement Noise [N^2]	Motor Variability [N^2]	Total Noise [N^2]
**Ideal Force**	0.0078	0.13	0.14
**Actual Force**	0.23	0.29	0.52

In particular, we calculated the measurement noise from our sensor specifications, and the intrinsic motor variability from subjects' data during the asymptotic region of learning using the mid-movement force metric described in the main text. Thus, the ratio of total noise between the two signals was around 0.26. Using this noise ratio, we calculated the Deming slope as:

$$b_{Deming} = \frac{\lambda Q_Y - Q_X + \sqrt{\left(Q_X - \lambda Q_Y\right)^2 + 4\lambda Q_{XY}^2}}{2\lambda Q_{XY}}$$

where λ is the noise ratio (0.26), $Q_X = \sum_i (x_i - \bar{x})^2$, $Q_Y = \sum_i (y_i - \bar{y})^2$, and $Q_{XY} = \sum_i (x_i - \bar{x})(y_i - \bar{y})$ (Wakkers et al. 1975). Since the actual force (*y*) is much noisier than the ideal force (*x*), the unbiased Deming slope (black curve) will be much closer to the *y*-onto-*x* slope (blue curve) than the *x*-onto-*y* derived slope, as seen in Figure S2. Thus, the adaptation indices we use are very close to the unbiased Deming slope.

An alternative measure for adaptation can be calculated by regressing the first principal component of the actual force pattern onto the first principal component for the ideal force pattern. The motivation for this approach is that this analysis may reduce the noisiness of the signals being regressed. We note that while the *y*-onto-*x* regression minimizes the *y*-error, the *x*-onto-*y* regression minimizes the *x*-error, and the Deming regression minimizes a combination of these errors in a weighted fashion (with the weighting pre-determined by the ratio of noise in the two signals), PCA finds the line that minimizes its orthogonal error (Linnet 1998). Therefore, for PCA, the weighting is directly determined by the regression slope. Performing PCA on our dataset yields an adaptation curve which asymptotes at or above 1 (Figure S2, green curve), implying that subjects are essentially producing the ideal force pattern. However, Figure 3 in the main text shows that the averaged actual force pattern produced late in Task A (green curve) does not perfectly match up with the ideal force pattern (gray dashed curve), as implied by a PCA learning index of 1. Instead, the actual force is noticeably less than the ideal force, especially around the peak speed point. It is also worth noting that learning curves constructed using mid-movement force as a learning metric display asymptotic learning levels that also fall short of ideal performance (see Figure S4A). This suggests that the PCA method produces artifactually high regression coefficients in our data set because the error weightings it uses are inappropriate.

Interestingly, when we perform our regression analyses on force patterns averaged across subjects, we find that the *y*-onto-*x* slope (Figure S3, solid blue curve) remains essentially the same as when the regression was performed on individual trials for individual subjects, and then averaged across subjects (Figure S3, dashed blue curve). Note the y-range of the plot is from 0.6 to 1 – this is to facilitate distinguishing between the different traces. Averaging force patterns across subjects substantially reduces both measurement noise and subject-specific motor variability in our data, and brings the *x*-onto-*y*, Deming, and PCA slopes all much closer to the *y*-onto-*x* slopes. Note that the center of the new range of values is not shifted towards the center of the previous range (which was about 1, see Figure S2), but is instead shifted towards the lower-bound of the previous range – the *y*-onto-*x* curve. This suggests that our choice of the *y*-onto-*x* slope we used is not significantly biased by measurement noise and subject-specific motor variability in *x* (where *x* is the ideal force), and thus is a very reasonable metric to use to quantify adaptation.

Mid-movement force as an adaptation index

In addition to using the regression coefficients as adaptation indices, we also assessed adaptation by quantifying the amount of force associated with learning-related changes used to counteract the force-field (Figure S4). Since the lateral force required to oppose the force-field is greatest at the peak speed point, which is near the middle of the movement, we used the average mid-movement force as a secondary measure of the progression of adaptation. We defined the mid-movement force as the average force produced during a 250ms window centered at the movement's peak speed.

Alternative anterograde interference metrics

Metrics starting from Task B onset

There are several reasons why we used the zero-crossing point as the start of opposite-learning. As mentioned in the main text, evaluating learning from the first trial in Task B confounds retention of performance in Task A with

a reduced learning rate of Task B caused by prior exposure to Task A. By using the zero-crossing point, we are able to remove the confound caused by retention of Task A and thus evaluate *only* the reduced learning rate. This comparison between initial and opposite learning curves proceeding from the same performance level (i.e. zero) is analogous to the comparison between initial learning and relearning rates in the analysis of savings in the A_1BA_2 paradigm. If complete unlearning is not achieved before relearning begins, then analysis of the relearning curve will reflect both the retention of performance of Task A_1 and the increased learning rate caused by prior exposure to A_1, making it difficult to dissociate the contributions of the two factors.

Our decision to start evaluating interference following the zero-crossing point is also justified because certain metrics that began from Task B onset will actually show interference when there is none. To illustrate this point, imagine that we used just a single-process model to explain our findings instead of the two-process multi-rate model. Such a model would *not* predict interference (Figure S5). A model with a single exponential process would predict *exactly* the same rate of learning for initial learning of Task A as for opposite-learning of Task B, as can be seen in comparison of initial learning and opposite-learning curves once they have been properly aligned to the zero-crossing point (Figure S5). The zero-crossing-based AI metric we use in our paper properly indicates that no interference is occurring in this single-process model because the initial learning and opposite-learning curves are identical (Figure S6C, dotted gray line). However, we also looked at two measures of interference with respect to the initial trial of Task B: (1) the raw adaptation level after 50 trials of exposure to Task B, and (2) the change in adaptation from trial 1 to trial 50 of Task B. We chose to base these new metrics on 50 trials of exposure to Task B because we wanted these metrics to be sensitive to both the amount of time spent in Task B prior to the zero-crossing (between 5 and 34 trials) and the range over which we calculated our original AI metric (trials 1-25 following the zero-crossing point). Even though by definition there is *no* interference in the single-process model, the raw adaptation after 50 trials of Task B decreases as Task A duration increases, a pattern that could be interpreted as interference (Figure R8A, dotted gray line), whereas the change in adaptation over 50 trials shows an increase, a pattern that could be interpreted as a decrease in interference (Figure R8B, dotted gray line). As a result, these two metrics are inappropriate measures for characterizing interference because they show patterns that would appear consistent with interference or facilitation, even in cases in which no interference or facilitation occurs.

Nevertheless, we calculated these two metrics for our data set, in keeping with the reviewer's suggestion. Displayed in Figure S6A is the raw adaptation level after 50 trials of exposure to Task B (note that this raw adaptation level is rectified – the sign is flipped). Both the data and simulated metrics (dashed gray line, gray confidence intervals represent two-process multi-rate model predictions) express very similar patterns of interference to the single-process model's predictions, which we previously showed can falsely show interference. Furthermore, this raw adaptation level does not take into account the performance level at Task B onset. The 13-trial group starts from a much lower level of performance than the 112-trial, 230-trial, and 369-trial groups (see Figure 4A), meaning that these subjects do not have to overcome as much Task A retention as the other groups, which could explain why the 13-trial group is able to reach greater levels of Task B adaptation. This metric is therefore unable to dissociate the confounding contributions of retention of Task A performance and a slowed learning rate for Task B caused by prior exposure to Task A – and as a result, the interference effect, although present, is clearly muted.

When we look at adaptation after 50 trials *relative* to the initial performance in Task B (Figure S6B), the interference effect has now disappeared, both in the modeling results and the data. There may even be a subtle upwards trend, indicating that as Task A duration is increased, there is slightly *more* change in adaptation, which would be facilitation and not interference, as seen in the single-process model's predictions. However, the groups with longer Task A training achieved higher performance levels, and thus when Task B is begun, they have *higher* motor error with which to drive learning. This larger learning signal induces greater learning that is not necessarily related to a change in the learning rate, and could potentially overshadow any effects of a decreased learning rate. As a result, this metric is also unable to dissociate the effects of higher performance at Task B onset and a slowed learning rate. In summary, these two metrics which begin from Task B onset are inappropriate

measures of interference because they falsely show interference where there is none, and because they cannot dissociate retention of adaptation from a slower learning rate.

Time constant as an interference metric

As opposed to looking at average learning over a set number of trials (as we do in the main text) to evaluate interference, we could use a more direct way, such as by estimating the time constant of the opposite-learning curves. However, we used the current measure because we found it to be more robust to the effects of noise, which are considerable. For example, please see Figure S7 for analysis of the time constant as a metric for interference. We see that as Task A duration increases, the time constant also increases (Figure S7A: linear scale on the y-axis, Figure S7B: log scale on the y-axis), much like our current AI metric does (see Figure S7C for a replotting of our AI metric). This indicates that as Task A duration is increased, the groups with longer training in Task A have slower opposite-learning curves. However, even though the time constant metric can capture interference, it is a noisier measure than our area metric. In Figure S7D, we show the coefficients of variation (i.e. the ratio between standard deviation and mean) for these two metrics. Low coefficients of variation indicate lower levels of variability with respect to the mean. With the exception of the 13-trial group, the coefficient of variation is higher for the time constant metric than for the area metric. On average, the coefficient for variation for the time constant metric (dotted line) is 22% higher than that for the area metric (dashed line). This noisiness manifests itself in other ways, as well. When estimating the time constants for the individual opposite-learning curves, it was necessary to constrain our parameter space to prevent instabilities in the fitting routine. In particular, we used the following form:

$$y = A \cdot \left(1 - e^{-n/\tau}\right)$$

where we constrained A to be between 0 and 1, and τ to be between 0 and 100 trials. Without implementing these bounds, our least squares quadratic routine yielded unreasonable parameter estimates (e.g. some individual curves had A's and τ's larger than 10^{10}, completely distorting the mean). In summary, time constants are, for our data, much noisier (less reliable) measures. Their values must be manually constrained to obtain reasonable group averages, and even with such constraints (which are hard to justify from first principles), the noise levels are still higher, as shown in Figure S7D.

Possible choices for zero-crossing point

After Task B onset, two of the groups (230-trial and 369-trial groups) had their adaptation go below zero, return above zero, and then go below zero again. In the main text, we chose the second zero-crossing as the beginning of the opposite-learning curves (Figure 4C and S8A for a smoothed version). The unsmoothed opposite-learning curves are shown in the middle panel of Figure S8A, and the associated AI metrics are displayed in the bottom panel of Figure S8A. We also found the opposite-learning curves and AI metrics by choosing to use the average of the first and second zero-crossings as the beginning of the opposite-learning curves. These results are shown in Figure S8B. Note that our simulation predictions can explain the AI pattern calculated if using the second zero-crossing point (R^2=0.91), as well as if using the average zero-crossing (R^2=0.86) as the beginning of the opposite-learning curves.

Alternative explanations for anterograde interference

One model that could provide an alternative explanation for anterograde interference besides the multi-rate model is the memory trace model as described in Anderson et al. 1999. In this framework, overall strength of a memory can be thought of as the summed contributions of individual traces. A new trace is introduced every stimulus presentation, and as training proceeds, the traces stack on top of each other, with the strength of each trace

obeying a power law decay function, which has been proposed by numerous groups (Wickelgren 1972, Rubin and Wenzel 1996, Wixted and Carpenter 2007). Specifically, $S=\sum_{i=1}^{n} k\cdot t_i^{-d}$, where S is the overall memory strength, k is the initial strength of an individual trace, t_i is the time that has passed since the ith occurrence of the stimulus, d is the constant parameter characterizing the decay rate decay, and the summation is over the total number of stimulus presentations. There are four important differences between this memory trace model and our multi-rate model. First, according to the memory trace model, each time a stimulus is presented, a single trace is added to the memory strength. However, according to the multi-rate model, two traces are added simultaneously – a fast memory trace and a slow memory trace. Second, according to the memory trace framework, each added trace has the same initial strength (i.e. k), whereas in the multi-rate model, the initial values of the fast and slow traces are each proportional to the motor error, and thus to each other. Third, the memory trace framework states that each trace obeys power law decay, whereas the multi-rate model states that the fast and slow traces both decay exponentially. Fourth, in the memory trace model, the traces decay as a function of time, whereas in the multi-rate model, the fast and slow traces decay as a function of trial. Writing the learning rule of our multi-rate model in the same form as the memory trace learning rule yields:

$$S=\sum_{i=1}^{n}\left[B_{fast}error_i A_{fast}^{m_i-1}+B_{slow}error_i A_{slow}^{m_i-1}\right]$$

where B_{fast} and B_{slow} are the learning rates, A_{fast} and A_{slow} are the retention rates of the fast and slow processes, respectively, m_i is the number of trials since the ith presentation, and $error_i$ is the difference between the desired motor output and the actual motor output (i.e. the summation of the fast process and slow process) on trial i. This representation of learning is equivalent to the learning equations for the fast and slow processes given in the Methods section.

In implementing the memory trace framework for our Task A/Task B learning paradigm, we assigned a $+k$ initial value to all traces added during Task A, and a $-k$ initial value to all traces added during Task B (Figure S9A). Using the learning rule $S=\sum_{i=1}^{n} k\cdot t_i^{-d}$, with k=0.38, d=1, and an inter-trial interval of 5secs, we find that the memory trace framework predicts that opposite-learning is slower than initial learning, or anterograde interference (Figure S9B). However, one problem with this framework is that since memory strength is encoded by superposition of traces with identical initial values, there is no intrinsic upper bound for the memory strength. For instance, if the value of k is too high, then the memory strength, which has been used to represent the percentage of correct recall (Anderson and Schooler 1991), could exceed 100%. To account for this lack of an upper bound, data and experiment structure (such as inter-trial interval, ITI) are needed to estimate parameters that can ensure that the memory strength remains within a reasonable range, such as between 0% and 100%. This dependence on ITI is made more explicit if we rewrite the learning rule as:

$$S=\sum_{i=1}^{n} k\cdot t_i^{-d}=\sum_{i=1}^{n} k\cdot\left(T_{ITI}\cdot m_i\right)^{-d}=\sum_{i=1}^{n}\left(k\cdot T_{ITI}^{-d}\right)\left(m_i^{-d}\right)$$

where i is the trial number, $t_i=T_{ITI}\cdot m_i$, T_{ITI} is the inter-trial interval, and m_i is the number of trials since the ith presentation. The computations required to determine the appropriate level of k are non-trivial, and are influenced by information that is available only *after* training is complete, such as the total number of training trials. For example, if training is only for a short number of trials, then k can be higher than if there are a large number of training trials.

Note that if the training paradigm has non-constant ITIs, k would have to be recalibrated every time ITI changed in order to ensure that the memory strength remains in the reasonable range. This poses a risk however, because estimation of ITI requires at least two trials. If a subject is expecting a long ITI (and has accordingly set k to be higher) but then receives a short ITI, the inappropriately high k value could send the memory strength over 100%.

In summary, compensation for the lack of an intrinsic upper bound for memory strength in this framework requires non-trivial computations that are only tractable *after* training is complete. One method to impose an upper bound, however, is to treat the traces as a *gain* on an error term, where error is defined as the difference between actual strength and desired strength. Thus, as the actual strength approaches the upper bound set by the desired strength, error approaches zero, leading to smaller and smaller changes in memory strength. This altered learning rule might take the form:

$$S = \sum_{i=1}^{n} \left(k \cdot T_{ITI}^{-d} \right) \cdot error_i \cdot \left(m_i^{-d} \right)$$

Note that this equation is quite similar to the two-process, multi-rate model discussed in the current paper, but written in a slightly different form.

$$S = \sum_{i=1}^{n} B_{fast} \cdot error_i \cdot \left(A_{fast}^{m_i - 1} + \frac{B_{slow}}{B_{fast}} A_{slow}^{m_i - 1} \right)$$

The decay terms of these two models, $\left(m_i^{-d} \right)$ and $\left(A_{fast}^{m_i - 1} + \frac{B_{slow}}{B_{fast}} A_{slow}^{m_i - 1} \right)$, differ because the memory trace framework expresses power law decay, whereas the two-process, multi-rate model expresses two-exponential decay. However, the decay profiles as a function of trial are quite similar (Figure S10), where d=0.82, and the parameters for the multi-rate decay are given in the Methods section. This similarity is not surprising given that it is well-known that power law decay is tantamount to the simultaneous decay of multiple timescales within the system, which can alternatively be expressed as the combination of multiple exponential decay functions (Bochud and Challet 2007). Consequently, the two-exponential decay in our multi-rate model can be thought of as a subset of power law decay. Interestingly, however, Rubin et al. showed that when attempting to characterize the decay of declarative memories, a function containing just *two* exponential decay functions actually explains their data *better* than the power law decay functions they tested (Rubin et al. 1999), which notably corresponds to our two-process multi-rate model.

Effect of Task A duration and magnitude of Task A perturbation on AI metric

In the main text, we argue that it is the level of the slow process that determines the amount of AI, and because our model is linear, we expect that the level of the slow process and thus the amount of interference should be linearly related to the strength of the force-field. To demonstrate this, we simulated the effect on AI of varying the magnitude of the Task A perturbation along with Task A duration (Figure S11). When the magnitude of Task A is at 100% strength, the predicted AI metrics are identical to those displayed in Figure 5C (gray dotted line). However, as the magnitude of Task A is reduced, the predicted amounts of AI also decrease for all Task A durations. Note that the amount of interference falls off monotonically with decreases in the Task A magnitude (i.e. the initial force-field strength). This reduction in interference reflects the decreased levels of the slow process achieved during Task A, and highlights the dependence that AI has on the magnitude of the Task A force-field.

Multi-rate model parameters fit to current data set

Parameters	Previous Data Set (Smith et al. 2006)	Previous Data Set 95% CI	Current Data Set
A_{fast}	0.6	[0.37,0.70]	0.55
B_{fast}	0.2	[0.10,0.35]	0.14
A_{slow}	0.9924	[0.9904,0.9943]	0.9967
B_{slow}	0.02	[0.011,0.023]	0.021

The 95% confidence intervals for the parameter estimates from the previous data set were determined by a bootstrapping routine where 1000 different subsets of the data were fit with the 4-parameter, two-process, multi-rate model (Smith et al. 2006). Then, each parameter space was taken independently, sorted, and the 25th and 975th values were chosen as the 95% confidence bounds for that parameter. Note that this analysis does not keep together the parameter sets found for particular subsets of the data.

The parameters from the current data set were obtained by fitting both the overall learning curves and the AI metric data, and are all within the 95% confidence intervals of the previous data set, with the exception of A_{slow}. This may be the case because our current experiment was not optimally designed to estimate the parameter values. In contrast, the previous experimental design contains an experimental manipulation that allows for direct measurement of the A_{slow} parameter. Specifically, the Smith et al. report contained an extended period of error-clamp trials during which error-driven learning was largely eliminated, and the fast process quickly decayed away, leaving only the decay of the slow process as the contributor to learning changes.

Bibliography for Text S1:

Anderson JR, Schooler LJ (1991) Reflections of the Environment in Memory. Psychol Sci 2: 396-408.

Anderson JR, Fincham JM, Douglass S (1999) Practice and retention: a unifying analysis. J Exp Psychol Learn Mem Cogn 25: 1120-1136.

Berkson J (1950) Are There Two Regressions? J Am Stat Assoc 45: 164-180.

Bochud T, Challet D (2007) Optimal approximations of power laws with exponentials: application to volatility models with long memory. Quant Financ 7: 585 - 589.

Cornbleet PJ, Gochman N (1979) Incorrect least-squares regression coefficients in method-comparison analysis. Clin Chem 25: 432-438.

Linnet K (1998) Performance of Deming regression analysis in case of misspecified analytical error ratio in method comparison studies. Clin Chem 44: 1024-1031.

Rubin DC, Hinton S, Wenzel A (1999) The precise time course of retention. J Exp Psychol Learn 25: 1161-1176.

Rubin DC, Wenzel AE (1996) One hundred years of forgetting: A quantitative description of retention. Psychol Rev 103: 734-760.

Smith MA, Ghazizadeh A, Shadmehr R (2006) Interacting Adaptive Processes with Different Timescales Underlie Short-Term Motor Learning. PLoS Biol 4: e179.

Wakkers PJM, Hellendoorn HBA, De Weegh GJO, Heerspink W (1975) Applications of statistics in clinical chemistry: A critical evaluation of regression lines. Clin Chim Acta 64: 173-184.

Wickelgren WA (1972) Trace resistance and the decay of long-term memory. J Math Psychol 9: 418-455.

Wixted JT, Carpenter SK (2007) The Wickelgren Power Law and the Ebbinghaus Savings Function. Psychol Sci 18: 133-134.
